# Biologic Therapy and Surgical Management in Crohn’s Disease: Postoperative Outcomes and Biologic Management Patterns in a Retrospective Cohort Study

**DOI:** 10.3390/medicina62050917

**Published:** 2026-05-08

**Authors:** Constantin-Alexandru Petraru, Tudor Stroie, Doina Istratescu, Dan Pitigoi, Corina Gabriela Meianu, Rucsandra Ilinca-Diculescu, Mircea Diculescu

**Affiliations:** 1 Faculty of Medicine, “Carol Davila” University of Medicine and Pharmacy, 050474 Bucharest, Romania; alexandru.petraru92@gmail.com (C.-A.P.); corina_meianu@yahoo.com (C.G.M.); ridiculescu@yahoo.com (R.I.-D.); mmdiculescu@yahoo.com (M.D.); 2 Department of Gastroenterology, Fundeni Clinical Institute, 022328 Bucharest, Romania; doina.proca08@gmail.com (D.I.); pitigoidancristian@yahoo.com (D.P.)

**Keywords:** Crohn’s disease, intestinal resection, fecal diversion, biologic therapy, biologic intensification, postoperative outcomes, treatment-refractory disease

## Abstract

*Background and Objectives*: The therapeutic role of surgery in Crohn’s disease has evolved in the era of advanced biologic therapies, particularly in patients with complex and treatment-refractory disease. This study aimed to evaluate the relationship between preoperative biologic exposure and surgical outcomes, with a focus on predictors of more extensive surgical procedures, postoperative biological response, and postoperative biologic management. *Materials and Methods*: We conducted a retrospective cohort study including 60 patients with Crohn’s disease who underwent CD-related surgical interventions between January 2011 and December 2024. Clinical, surgical, and therapeutic data were collected. Combined resection procedures were defined as intestinal resections associated with additional surgical interventions. Postoperative biological response was defined as an exploratory composite endpoint reflecting the simultaneous normalization of hemoglobin, serum albumin, and C-reactive protein at six months. Statistical analyses, including univariable and multivariable methods, were performed. *Results*: Combined resection procedures were associated with advanced disease, particularly penetrating phenotypes and intra-abdominal sepsis, and with more frequent postoperative biologic intensification (OR 5.56, 95% CI: 1.05–29.57, *p* = 0.044). Postoperative biologic management included maintenance and intensification strategies (initiation or switching of biologic therapy). At six months, postoperative biological response was achieved in 20.7% of patients (12/58). No significant associations were observed between biological response and preoperative anti-TNF exposure or postoperative biologic intensification. Despite the relatively low rate of complete biological normalization, hemoglobin and albumin normalization were observed in 79.3% and 69.0% of patients, respectively, while the median fecal calprotectin decreased from 820 µg/g preoperatively to 130 µg/g at follow-up. Endoscopic remission was observed in 47.6% of patients with available SES-CD assessment. *Conclusions*: In patients with complex Crohn’s disease, surgical intervention remains an essential component of multidisciplinary management. While complete postoperative biological normalization was achieved in a limited proportion of patients, surgery was associated with consistent improvements in inflammatory and nutritional parameters. Further prospective studies are needed to better define predictors of postoperative recovery and to clarify the role of surgery within modern treatment algorithms.

## 1. Introduction

The therapeutic landscape of Crohn’s disease (CD) has undergone a profound transformation over the last two decades, driven by the rapid expansion of the medical armamentarium and a parallel shift in surgical philosophy. Historically, surgery was viewed as a “last resort” reserved for patients who had failed medical therapy or developed life-threatening complications. However, contemporary multidisciplinary strategies have repositioned surgical intervention as an integral component of disease management, with the potential to influence disease course when appropriately timed [1,2]. This shift in paradigm was largely catalyzed by the landmark LIR!C study, which demonstrated that for patients with limited, non-stricturing ileocecal disease, early laparoscopic ileocecal resection serves as a safe and cost-effective alternative to anti-TNF agents [3]. These findings have been further supported by long-term follow-up data confirming the sustained benefits of early resection [4] and further validated by large-scale multicenter studies investigating outcomes in complicated ileal disease [5]. These data have contributed to the emerging concept of early surgical intervention as a valid strategy in selected patients with Crohn’s disease, particularly in the context of ileal involvement and limited disease extent.

Despite the introduction of advanced therapies, including integrin inhibitors, interleukin-12/23 inhibitors, and Janus kinase inhibitors, the challenge of medical refractoriness persists [1]. In contemporary practice, many patients reaching the operating table are biologically exhausted, having failed multiple lines of advanced therapy (such as biologic and targeted agents) and often presenting with impaired nutritional status [6,7]. In this context, clinical refractoriness, defined as a documented lack of response to optimized medical treatment, has emerged as a major indication for surgery in specialized tertiary centers.

Recent advances in Crohn’s disease management have expanded the therapeutic landscape with the introduction of newer biologic agents targeting interleukin pathways (IL-12/23p40 and IL-23p19 inhibitors) and small molecule therapies such as Janus kinase inhibitors [8,9,10]. In parallel, treatment strategies have evolved toward a treat-to-target approach, aiming for objective control of inflammation and prevention of long-term complications [1,11,12]. However, access to these therapies has been heterogeneous across healthcare systems, with differences in availability and timing of adoption reported across regions, including parts of Eastern Europe, where the integration of newer therapeutic agents into routine clinical practice has occurred gradually [13,14].

As the clinical burden of these patients increases, traditional frameworks like the Montreal classification remain essential for baseline phenotyping but do not fully capture intraoperative disease complexity [15]. In this context, we focused on the surgical approach by identifying combined resection procedures, defined as intestinal resections associated with additional surgical maneuvers such as stoma formation, intra-abdominal abscess drainage, or stricturoplasty, reflecting a more advanced disease presentation, frequently associated with penetrating phenotypes and increased preoperative inflammatory burden [2,7].

Furthermore, the identification of clinical factors associated with such surgical strategies is relevant for optimizing perioperative management. Surgical resection in Crohn’s disease can be understood as a means of reducing the overall inflammatory burden by removing macroscopically diseased segments, often followed by improvements in biological parameters such as hemoglobin, serum albumin, and C-reactive protein. Such changes may provide a favorable context for postoperative medical strategies [7,16,17].

The primary objective of this study was to evaluate the relationship between preoperative biologic exposure and surgical outcomes, with a focus on predictors of combined resection procedures, postoperative biological response, and postoperative biologic management strategies in the current era of advanced therapeutics. Thus, we aim to provide a more nuanced understanding of the interaction between medical refractoriness and surgical outcomes in Crohn’s disease [18].

## 2. Materials and Methods

### 2.1. Study Design and Patient Selection

This retrospective observational cohort study was conducted at the Fundeni Clinical Institute, a tertiary referral center in Bucharest, Romania, between January 2011 and December 2024. The study was conducted in accordance with the Declaration of Helsinki and approved by the Ethics Committee of the Fundeni Clinical Institute, Bucharest, Romania (initial approval: 13 July 2023; updated approval: 11 February 2026). Adult patients (≥18 years) with a confirmed diagnosis of Crohn’s disease (CD) according to ICD-10 criteria who underwent an index surgical intervention at our institution were eligible for inclusion. The index surgery was defined as the first CD-related surgical procedure performed at our center during the study period. Procedures not directly related to active Crohn’s disease, such as isolated stoma reversal or incisional hernia repair, were not considered index surgeries but were recorded as subsequent interventions during follow-up. All data were extracted from institutional electronic medical records, operative reports, and discharge summaries.

The analyzed cohort does not reflect the total surgical volume of the institution. Patient inclusion was restricted to cases managed within a single gastroenterology unit of the institution, in order to ensure consistency in preoperative evaluation, medical management, and postoperative follow-up. Patients were included if sufficient perioperative and follow-up data were available to allow for reliable characterization of the primary study variables. This approach resulted in a well-defined and clinically homogeneous cohort.

### 2.2. Clinical Phenotype and Laboratory Variables

Disease phenotype was categorized according to the Montreal classification: age at diagnosis was defined as A1 (≤16 years), A2 (17–39 years), and A3 (≥40 years); disease location was recorded as L1 (ileal), L2 (colonic), and L3 (ileocolonic), with upper gastrointestinal involvement (L4) recorded when present; and disease behavior was classified as B1 (non-stricturing/non-penetrating), B2 (stricturing), or B3 (penetrating). Perianal disease was recorded using the Montreal “p” modifier when present.

Laboratory parameters including hemoglobin, serum albumin, ferritin, C-reactive protein (CRP), and fibrinogen were recorded within 30 days prior to surgery and reassessed at six months after surgery. Anemia was categorized as mild (10–12 g/dL), moderate (7–10 g/dL), or severe (<7 g/dL). Hypoalbuminemia was classified as mild-to-moderate (2.5–3.5 g/dL) or severe (<2.5 g/dL).

Systemic inflammation was assessed using CRP (normal ≤5 mg/L, mild/moderate 5–50 mg/L, or markedly elevated >50 mg/L) and fibrinogen levels (normal 200–400 mg/dL, moderate 400–600 mg/dL, or severe >600 mg/dL). Fecal calprotectin and ferritin values were recorded when available within three to six months before surgery and at the six-month follow-up.

### 2.3. Preoperative Medical Therapy

Preoperative treatment exposure was defined as any therapy administered within six months prior to surgery, including systemic corticosteroids, immunomodulators, and biologic agents.

To evaluate the degree of medical refractoriness, the complete biologic treatment history was recorded, including the number of prior biologic lines (ranging from 0 to 4). Preoperative biologics were stratified by therapeutic class, distinguishing anti-TNF agents (infliximab, adalimumab) from newer biologic or targeted therapies (ustekinumab, vedolizumab, and Janus kinase inhibitors). This distribution should be interpreted in the context of the study period, during which access to newer biologic therapies in Romania was progressively introduced, with anti-TNF agents representing the predominant advanced treatment options in the earlier years of the cohort.

Postoperative treatment strategies were categorized as biologic intensification, defined as initiation of a biologic agent in biologic-naive patients or switching to a different biologic therapy after surgery, and non-intensification, defined as maintenance of the same biologic therapy or absence of biologic treatment after surgery. The biologic therapy administered at 6 and 12 months after surgery was recorded to evaluate postoperative therapeutic evolution. Postoperative therapeutic variables, including biologic intensification, were analyzed as postoperative correlates of surgical outcomes rather than as preoperative predictors.

### 2.4. Surgical Procedures and Operative Characteristics

Index surgery was defined as the first Crohn’s disease-related surgical intervention performed in our center during the study period, regardless of prior surgical history. For each index surgery, operative urgency was classified as elective or urgent, and the surgical approach was recorded as laparoscopic or open (laparotomy). Previous abdominal surgical history was recorded and analyzed as a categorical variable.

Indications for surgery included medically refractory disease, stricturing complications (ileal, colonic, or rectal strictures), penetrating disease manifestations including intra-abdominal or enterocutaneous fistulas, intra-abdominal abscesses, and acute complications such as bowel obstruction.

Recorded surgical procedures included ileocecal resections, small-bowel resections, segmental colonic resections, hemicolectomies, subtotal or total colectomies, proctocolectomies, and rectal or rectosigmoid resections. Non-resectional procedures such as intra-abdominal abscess drainage and diverting stoma creation were documented as separate variables. Organ-preserving procedures such as stricturoplasty were also recorded descriptively.

Combined resection procedures were defined as intestinal resections associated with additional surgical maneuvers, including stoma formation, intra-abdominal abscess drainage, or extended resections (such as total or subtotal colectomy and rectal resections), as well as procedures incorporating bowel-sparing techniques such as stricturoplasty.

To better characterize the longitudinal surgical history of the patients, cumulative surgical burden was assessed descriptively based on both prior surgical history and the need for reintervention during follow-up. Patients were categorized as requiring a single surgical procedure or at least one additional surgery. Subsequent interventions were further characterized according to their indication (such as stoma reversal or disease-related reintervention) and by the number of procedures performed.

### 2.5. Postoperative Outcomes and Definitions

Postoperative complications were classified according to timing as early (≤30 days) or late (>30 days). Recorded complications included infectious events (including Clostridioides difficile infection), thrombotic events, acute kidney injury, gastrointestinal bleeding, postoperative ileus, anastomotic leakage or dehiscence, stomal prolapse, and evisceration. Management strategies were documented, distinguishing between conservative treatment and the need for surgical reintervention within 30 days, which was recorded as a separate outcome variable.

A composite outcome termed postoperative biological response was defined as the simultaneous normalization of hemoglobin (absence of anemia according to predefined thresholds), serum albumin (>3.5 g/dL), and C-reactive protein (≤5 mg/L) at six months after surgery.

### 2.6. Endoscopic Assessment

Postoperative endoscopic disease activity was evaluated, when available, at approximately six months using the Simple Endoscopic Score for Crohn’s Disease (SES-CD), with disease activity categorized as remission (0–2), mild (3–6), moderate (7–15), or severe (≥16). In cases where SES-CD values were not explicitly documented in the original endoscopy reports, the score was retrospectively derived from the available endoscopic descriptions when sufficient information was present. For patients who underwent ileocecal resection, postoperative recurrence was additionally assessed using the Rutgeerts score, with i0–i1 considered indicative of low-risk postoperative recurrence. Owing to the retrospective design, these endoscopic scores were subject to substantial missingness (65% for SES-CD and 83.3% for Rutgeerts) and were therefore reported descriptively without inclusion in the primary inferential analyses.

### 2.7. Statistical Analysis

Statistical analysis was performed using the SPSS Statistics version 20.0 (IBM Corp., Armonk, NY, USA). Continuous variables are presented as medians (interquartile range, IQR). Categorical variables are reported as frequencies and percentages, with corresponding 95% confidence intervals (95% CI).

Univariate analysis of categorical variables was performed using Fisher’s exact test, particularly for comparisons involving small subgroups (e.g., stoma formation, surgical approach, combined resection procedures, and postoperative outcomes).

To identify independent predictors of surgical outcomes, variables with *p* < 0.1 in univariate analysis and considered clinically relevant were eligible for inclusion in multivariable logistic regression models. To reduce the risk of overfitting given the limited sample size, the number of variables included in the final models was restricted. The results of the multivariable analysis are reported as regression coefficients (B), Wald statistics, and odds ratios (OR) with 95% confidence intervals (CI). A *p*-value < 0.05 was considered statistically significant for all final analyses. Analyses were performed using available-case analysis without imputation of missing values.

## 3. Results

### 3.1. Baseline Characteristics

A total of 60 patients with Crohn’s disease (CD) who underwent surgical intervention were included in the study. The cohort was characterized by a relatively young age profile, with a median age at surgery of 32.5 years (IQR 25.3–41.0) and a predominance of complicated disease behavior. Complicated disease behavior was observed in the vast majority of patients, with stricturing (B2) and penetrating (B3) phenotypes accounting for 96.7% (*n* = 58) of cases within the Montreal classification, while the perianal modifier (p) was present in 20.0% (*n* = 12) of patients. This disease severity was further compounded by a substantial cumulative operative burden, as 45.0% (*n* = 27) of the cohort had undergone at least one prior abdominal or perianal surgery before the index procedure. Baseline demographic and phenotypic distributions are summarized in Table 1.

Preoperative objective assessments indicated a high systemic inflammatory load and significant nutritional depletion. Imaging confirmation (CT, MRI, or ultrasound) was nearly universal (95.0%, *n* = 57). Among patients with available endoscopic data (*n* = 40), moderate-to-severe endoscopic activity (SES-CD ≥ 7) was observed in 25 patients, corresponding to 62.5% of those with endoscopic assessment and 41.7% of the overall cohort. Preoperative fecal calprotectin, available for 26.7% (*n* = 16) of patients, was consistent with high mucosal inflammatory activity, with a median value of 820 µg/g. This inflammatory burden was also reflected in the laboratory profile, where the median C-reactive protein was 37.0 mg/L, and 56.9% (*n* = 33) of patients presented with elevated CRP levels (>5 mg/L). Hematological and nutritional depletion were also common, with 39.7% (*n* = 23) of patients presenting with anemia and 25.9% (*n* = 15) with hypoalbuminemia. Laboratory parameters were not available for all patients; therefore, percentages were calculated based on available data for each variable.

The cohort’s medical history was characterized by extensive prior therapeutic exposure. At the time of surgical referral, 75.0% (*n* = 45) of patients were receiving ongoing medical therapy, and 68.3% (*n* = 41) had been exposed to advanced therapies. Anti-TNF agents predominated (46.7%, *n* = 28), while a substantial proportion of patients (21.7%, *n* = 13) had been treated with newer biologics such as ustekinumab or vedolizumab. Notably, 21.7% (*n* = 13) of patients were considered clinically biologically exhausted, having failed two or more lines of advanced therapy (up to four lines) prior to the index surgery, as detailed in Table 2.

This profile is consistent with a cohort with advanced structural disease and significant systemic inflammatory burden, providing the clinical context for the surgical strategies and postoperative outcomes analyzed in this study.

### 3.2. Surgical Characteristics

#### 3.2.1. Surgical Indications and Operative Procedures

Surgical intervention was primarily elective (73.3%, *n* = 44), while the remaining 26.7% (*n* = 16) of procedures were performed in an urgent setting. Surgical indications reflected a combination of structural disease complications and clinical disease activity. Among urgent procedures, intestinal obstruction was the most frequent clinical scenario (56.3%, *n* = 9), followed by intra-abdominal septic complications, including abscess formation (43.8%, *n* = 7). From a clinical perspective, medically refractory disease was observed in 81.7% (*n* = 49) of cases, while signs of active inflammatory activity were present at the time of surgery in 90.0% (*n* = 54) of the cohort.

Within the Montreal classification, stricturing disease (B2 phenotype) was present in 56.7% (*n* = 34) of patients, while penetrating disease (B3 phenotype) was observed in 40.0% (*n* = 24).

Among stricturing manifestations, ileal strictures were the most common (61.7%, *n* = 37), followed by colonic (21.7%, *n* = 13) and rectal strictures (3.3%, *n* = 2). The clinical impact of stricturing disease was reflected by obstructive episodes, documented in 16.7% (*n* = 10) of patients. Long or multiple strictures were identified in 41.7% (*n* = 25) of patients, while short strictures (<5 cm) were observed in 38.3% (*n* = 23). Penetrating disease manifestations were also highly prevalent, including intra-abdominal abscesses in 38.3% (*n* = 23) and intra-abdominal or enterocutaneous fistulas in 33.3% (*n* = 20) of patients. Perianal fistulas were present in 13.3% (*n* = 8) of cases at the time of surgery.

Regarding the surgical approach, 53.3% (*n* = 32) of procedures were performed laparoscopically, while the remaining 46.7% (*n* = 28) were performed using an open surgical approach. Resection with primary anastomosis was achieved in 56.7% (*n* = 34) of patients, while 43.3% (*n* = 26) required fecal diversion, including ileostomy in 30.0% (*n* = 18) and colostomy in 13.3% (*n* = 8).

In terms of surgical procedures, ileocecal resection was the most frequently performed intervention (51.7%, *n* = 31), followed by right hemicolectomy (15.0%, *n* = 9), segmental colonic resection (10.0%, *n* = 6), left hemicolectomy (10.0%, *n* = 6), and isolated small bowel enterectomy (8.3%, *n* = 5). More extensive resections included total colectomy or proctocolectomy in 8.3% (*n* = 5), subtotal colectomy in 1.7% (*n* = 1), and rectal or rectosigmoid resections in 8.3% (*n* = 5).

A substantial proportion of patients required resections associated with additional surgical maneuvers, such as stoma formation or abscess drainage, reflecting the overall procedural burden of this cohort. Organ-sparing procedures were uncommon, with stricturoplasty performed in 3.3% (*n* = 2), including one Heineke–Mikulicz and one Michelassi procedure. Isolated abscess drainage was required in only 5.0% (*n* = 3) of patients, while in most cases intra-abdominal septic complications were managed in conjunction with bowel resection.

Complete operative characteristics are summarized in Table 3, while detailed anatomical disease features and procedural distribution are presented in Supplementary Table S1.

#### 3.2.2. Surgical Approach and Combined Resection Procedures

Surgical management was evaluated in terms of both operative approach and the need for additional intraoperative procedures, reflecting the overall surgical strategy in this cohort.

To identify clinical and biological factors associated with the surgical approach, we performed a univariate analysis comparing laparoscopic (*n* = 32) and open surgery (*n* = 28). A predefined set of clinically relevant variables was evaluated, including operative urgency, disease phenotype, and laboratory markers. Several factors were associated with an increased likelihood of laparotomy: surgical urgency (39.3%, *n* = 11 vs. 15.6%, *n* = 5; *p* = 0.047), intra-abdominal abscesses (53.6%, *n* = 15 vs. 25.0%, *n* = 8; *p* = 0.034), and preoperative hypoalbuminemia (39.3%, *n* = 11 vs. 13.3%, *n* = 4; *p* = 0.036). Non-significant trends toward an open approach were also observed for intra-abdominal fistulas (46.4%, *n* = 13 vs. 21.9%, *n* = 7; *p* = 0.058) and the penetrating (B3) phenotype (53.6%, *n* = 15 vs. 28.1%, *n* = 9; *p* = 0.065). Conversely, previous abdominal surgeries (*p* = 0.799) and multiple or long-segment strictures (*p* = 0.439) were not associated with the surgical approach.

To identify independent predictors of laparotomy, variables reaching the significance threshold in the univariate screening (*p* < 0.1) were entered into a multivariate logistic regression model. The analysis identified preoperative hypoalbuminemia and intra-abdominal abscesses as independent factors associated with laparotomy. Specifically, hypoalbuminemia increased the odds of laparotomy by 4.46-fold (OR = 4.463, 95% CI: 1.127–17.672, *p* = 0.033), while the presence of an abscess or collection increased the odds by 3.46-fold (OR = 3.465, 95% CI: 1.056–11.372, *p* = 0.040). Operative urgency remained of borderline significance in the final model (OR = 3.109, *p* = 0.090). These findings are summarized in Table 4.

Beyond the surgical approach, we evaluated combined resection procedures (as defined in the Methods) to assess whether similar factors were associated with the need for additional surgical maneuvers. Overall, 60.0% (*n* = 36) of patients underwent such procedures.

The univariate analysis identified several clinical and biological factors associated with combined resection procedures (Supplementary Table S2). The strongest associations were observed for penetrating disease features, including intra-abdominal abscesses (63.9% vs. 0%, *p* < 0.001) and intra-abdominal fistulas (52.8% vs. 4.2%, *p* < 0.001), which were significantly more frequent in patients undergoing these procedures.

Biological markers of severity, including preoperative hypoalbuminemia (*p* = 0.031) and markedly elevated CRP (>50 mg/L) (*p* = 0.001), were also significantly associated with combined resection procedures. In contrast, preoperative exposure to biologic therapy (*p* = 0.051) and the number of prior biologic lines (*p* = 0.236) were not significantly associated.

Notably, patients undergoing combined resection procedures were significantly more likely to require postoperative biologic intensification (47.2% vs. 16.7%, *p* = 0.026), defined as the initiation of a new biologic agent or a therapeutic switch.

To identify factors associated with combined resection procedures, variables with *p* < 0.1 in the univariate analysis were considered for multivariate modeling. Intra-abdominal abscesses were not included in the final model because they were present exclusively in the combined resection procedures group, resulting in complete separation and preventing stable estimation in logistic regression. Therefore, the final model included CRP, hypoalbuminemia, preoperative biologic exposure, intra-abdominal fistulas, and biologic intensification (Table 5), which was included for exploratory purposes given its postoperative nature and was not considered a determinant of intraoperative surgical decisions.

The analysis identified intra-abdominal fistulas as the primary independent predictor of combined resection procedures (OR = 22.93, 95% CI: 2.40–219.37, *p* = 0.007), suggesting a strong association, although the wide confidence interval indicates some degree of uncertainty in the estimate. In contrast, postoperative biologic intensification was more frequent in patients undergoing combined resection procedures (OR = 5.56, 95% CI: 1.05–29.57, *p* = 0.044) and remained statistically associated in the multivariable model. However, this finding reflects a postoperative correlate rather than a determinant of intraoperative surgical decisions. While inflammatory markers and nutritional status were significant in the univariate analysis, they did not retain statistical significance in the multivariate model. The results of the multivariate regression are summarized in Table 5.

#### 3.2.3. Fecal Diversion: Indications and Independent Predictors

In this cohort, fecal diversion was a major component of surgical management, performed in 43.3% (*n* = 26) of cases. Preoperative biologic therapy had been used in 68.3% (*n* = 41) of patients, most commonly anti-TNF agents (46.7%, *n* = 28), followed by newer biologics such as ustekinumab or vedolizumab (21.7%, *n* = 13). We evaluated whether preoperative anti-TNF exposure influenced the need for fecal diversion (Supplementary Table S3). However, anti-TNF therapy was not associated with the risk of either colostomy (25.0% vs. 11.5%, *p* = 0.264) or ileostomy (44.4% vs. 23.8%, *p* = 1.000), and no significant association was observed with overall fecal diversion (35.7% vs. 50.0%, *p* = 0.305) in our cohort.

While a diverting ileostomy was more frequent, occurring in 30.0% (*n* = 18) of patients, colostomy was required in 13.3% (*n* = 8). Given the different clinical implications of ileostomy and colostomy, these two forms of fecal diversion were analyzed separately. To explore the clinical associations of diversion, a univariate analysis was performed (Supplementary Table S3). Colostomy formation was associated with the presence of a perianal disease pattern (75.0% vs. 11.5%, *p* < 0.001), intra-abdominal fistulas (75.0% vs. 26.9%, *p* = 0.013), preoperative hypoalbuminemia (62.5% vs. 20.0%, *p* = 0.022), and the presence of abscesses (75.0% vs. 32.7%, *p* = 0.045).

When the two types of diversion were analyzed separately, distinct patterns emerged. For the colostomy subgroup, colostomy formation was more frequently observed in patients with a perianal disease phenotype (75.0% vs. 11.5%, *p* < 0.001) and in those undergoing left hemicolectomy as part of the surgical procedure (62.5% vs. 1.9%, *p* < 0.001). In contrast, ileostomy formation was significantly associated with the presence of intra-abdominal fistulas (55.6% vs. 23.8%, *p* = 0.034), while no other evaluated factors reached statistical significance.

To refine these findings, variables reaching a significance threshold of *p* < 0.1 in the univariate analysis were considered for inclusion in the multivariate model. Given the strong clinical and statistical overlap between intra-abdominal abscesses and fistulas, only intra-abdominal fistulas were retained in the final model to avoid collinearity. Therefore, the multivariate logistic regression included the perianal disease pattern, intra-abdominal fistulas, and preoperative hypoalbuminemia. The analysis identified perianal disease as the only variable that remained independently associated with colostomy formation (OR = 12.199, 95% CI: 1.653–90.031, *p* = 0.014), suggesting a strong association, although the confidence interval remains relatively wide. Although intra-abdominal fistulas and preoperative hypoalbuminemia were significant drivers in the univariate assessment, they did not maintain independent statistical significance when adjusted in the final model. These results, detailing the regression coefficients and significance levels, are summarized in Table 6.

In summary, these data reveal different patterns in the clinical associations observed for different types of fecal diversion. The perianal disease pattern was the only independent factor associated with colostomy formation (OR = 12.199, 95% CI: 1.653–90.031, *p* = 0.014). For ileostomy, a significant association was observed with intra-abdominal fistulas (*p* = 0.034), while no other variables reached statistical significance.

### 3.3. Postoperative Outcomes

#### 3.3.1. Postoperative Complications

Overall postoperative complications were documented in 23.3% (*n* = 14) of patients, including early (*n* = 12, 20.0%) and late (*n* = 2, 3.3%) events.

Detailed analysis of early postoperative complications (n = 12, 20.0%) showed that postoperative infectious complications were the most frequent events (*n* = 6, 10.0%), followed by Clostridioides difficile infections (*n* = 2, 3.3%). Postoperative ileus was observed in two patients (*n* = 2, 3.3%), occurring in association with one infectious complication in one case and one Clostridioides difficile infection in the other. Additional isolated complications included acute kidney injury (*n* = 1, 1.7%), anastomotic dehiscence (*n* = 1, 1.7%), postoperative hemorrhage (*n* = 1, 1.7%), and evisceration (*n* = 1, 1.7%). Early surgical reintervention within 30 days was required in 5.0% (*n* = 3) of the cohort. These cases included one anastomotic dehiscence requiring surgical revision with stoma creation, one postoperative hemorrhage requiring surgical hemostasis, and one postoperative evisceration managed by abdominal wall reconstruction.

Late complications were less frequent (*n* = 2, 3.3%) and consisted of one thrombotic event (*n* = 1, 1.7%) managed conservatively and one postoperative stenosis (*n* = 1, 1.7%) requiring endoscopic dilation.

Detailed complication categories and management outcomes are summarized in Table 7.

When stratified by preoperative anti-TNF exposure, early postoperative complications occurred in 21.4% (6/28) of patients receiving anti-TNF therapy and in 18.8% (6/32) of those not exposed to anti-TNF (Fisher’s exact test, *p* = 1.000). The non-anti-TNF group comprised both patients treated with newer biologic agents (*n* = 13, 21.7%) and those without any biologic exposure (*n* = 19, 31.7%). No statistically significant differences were observed between groups.

#### 3.3.2. Surgical Burden

Regarding the cumulative surgical burden, 53.3% (*n* = 32) of patients did not require any additional surgical intervention following the index procedure, while 46.7% (*n* = 28) underwent at least one subsequent surgery during follow-up.

Among patients requiring a second surgical intervention after the index procedure, the most frequent indication was the restoration of bowel continuity through planned stoma reversal (±additional resection), observed in 31.7% (*n* = 19) of the total cohort (67.9% of those undergoing reintervention). In contrast, 15.0% (*n* = 9) of patients required additional surgery related to an unfavorable disease course, including additional resections without restoration of bowel continuity or reoperations for postoperative complications.

A third surgical intervention was required in 10.0% (*n* = 6) of patients, while 3.3% (*n* = 2) underwent a fourth surgical procedure.

When all surgical interventions were considered, including prior surgical history, 61.7% (*n* = 37) of patients had undergone ≥2 surgical interventions.

Detailed surgical data are presented in Supplementary Table S5.

#### 3.3.3. Postoperative Medical Strategy

Postoperative medical management is presented in Table 8. The initial strategy at discharge involved biologic intensification in 35.0% (*n* = 21) of patients. This included the initiation of a first-line biologic in previously biologic-naive patients (23.3%, *n* = 14) or a therapeutic switch to a different biologic agent (11.7%, *n* = 7). In contrast, 48.3% (*n* = 29) of the cohort maintained their preoperative biologic therapy, while 3.3% (*n* = 2) received no postoperative biologic therapy. Strategy data at discharge were unavailable for 13.3% (*n* = 8) of patients. The primary agents utilized postoperatively were Adalimumab (33.3%, *n* = 20), Ustekinumab (26.7%, *n* = 16), and Infliximab (21.7%, n = 13).

Longitudinal follow-up showed changes in therapeutic strategy over time. At six months, 50.0% (*n* = 30) of patients remained on first-line biologic therapy, decreasing to 43.3% (*n* = 26) at 12 months. In parallel, the proportion of patients receiving second-line or higher biologic therapy increased from 28.3% (*n* = 17) at six months to 35.0% (*n* = 21) at one year. Additionally, 16.7% (*n* = 10) of patients required the introduction of an additional biologic agent during postoperative follow-up, as reported in Table 8.

#### 3.3.4. Postoperative Biological and Endoscopic Outcomes at 6 Months

At six months postoperatively, clinical and laboratory follow-up data were available for 58 patients (96.7% of the total cohort). A major exploratory endpoint of this study was the achievement of postoperative biological response, defined as the simultaneous normalization of hemoglobin (absence of anemia), serum albumin (>3.5 g/dL), and C-reactive protein (CRP ≤ 5 mg/L). Overall, 20.7% (*n* = 12) achieved postoperative biological response at six months.

As shown in Supplementary Table S4, the distribution of patients achieving postoperative biological response did not differ significantly between those undergoing combined resection procedures and those without (*p* = 0.746). This biological recovery was not significantly associated with postoperative biologic intensification (*p* = 0.513) or preoperative exposure to anti-TNF therapy (*p* = 0.349).

Overall clinical improvement across the cohort was observed. Hemoglobin normalization was achieved in 79.3% (*n* = 46) and serum albumin normalization in 69.0% (*n* = 40) of patients with available follow-up data. Inflammatory control was further supported by a marked reduction in median fecal calprotectin among patients with available data, decreasing from 820 µg/g preoperatively to 130 µg/g at six months, although these values were derived from a limited and partially overlapping subset of patients with available data.

Endoscopic reassessment was limited by the retrospective design, which explains the high proportion of missing endoscopic assessments. However, among the evaluated subset (SES-CD available in 21 patients), endoscopic remission (SES-CD 0–2) was observed in 47.6% (*n* = 10) of patients. Similarly, for the Rutgeerts score (available in 10 patients), low-risk postoperative recurrence (Rutgeerts i0–i1) was documented in 50.0% (*n* = 5) of assessed cases.

Despite overall clinical stabilization, persistent subclinical inflammatory activity (defined as CRP 5–50 mg/L) remained present in 32.8% of patients with available data, indicating residual inflammatory activity in a subset of patients.

## 4. Discussion

### 4.1. Phenotypic Severity of the Surgical Cohort

The present study examines the clinical profile of Crohn’s disease (CD) patients undergoing surgical intervention in a tertiary referral center, characterized by a young age distribution and a predominance of complicated disease phenotypes.

This study should be interpreted as a confirmatory, real-world analysis derived from a tertiary referral cohort in Romania. While the findings do not introduce novel mechanistic insights, they provide additional data from a geographical region that remains underrepresented in the current literature, where access to advanced therapies and treatment pathways has evolved differently over time.

The median age at surgery was 32.5 years, consistent with the well-recognized tendency of CD to affect individuals during early adulthood [2,19,20]. The cohort reflects an advanced stage of disease at the time of surgical referral. Long-term population-based studies have shown that many patients with CD eventually develop complicated disease behavior, including stricturing or penetrating phenotypes, often requiring surgical intervention during the disease course [21]. Nearly all patients exhibited complicated disease behavior, with stricturing (B2) and penetrating (B3) phenotypes accounting for 96.7% of cases. This distribution is consistent with the advanced disease stage typically observed in surgical cohorts, and likely reflects the cumulative effect of medically refractory disease over time [15,22,23,24].

Furthermore, the substantial cumulative operative burden observed in our cohort, where 45% of patients had already undergone at least one previous abdominal or perianal surgical intervention, highlights the progressive nature of the disorder managed in specialized centers. Repeated surgical interventions remain a well-recognized feature of the natural history of CD and reflect the chronic and relapsing course of the disease [25,26,27]. This high burden of prior surgical exposure further supports the interpretation that such centers predominantly manage patients with advanced and structurally complicated disease [2,28].

The therapeutic history of the cohort further illustrates the pronounced degree of medical refractoriness observed in the biologic era. More than two-thirds of patients had been exposed to advanced therapies prior to surgery, and over one-fifth had failed multiple biologic lines. This pattern suggests that in a substantial proportion of cases, surgical interventions occur after multiple therapeutic escalations have already been attempted, supporting the concept that surgery often represents a later step in the therapeutic management, rather than an early intervention [1,11].

These findings should also be interpreted in the context of an evolving therapeutic landscape, where newer agents such as interleukin-23 inhibitors and Janus kinase inhibitors are increasingly used. Limited access to these therapies during part of the study period may have influenced treatment pathways and contributed to the observed patterns of medical refractoriness.

Although preoperative biologic exposure was not significantly associated with the surgical outcomes analyzed in our cohort, a borderline association was observed for combined resection procedures (*p* = 0.051). This finding should be interpreted cautiously, but may still suggest that prior biologic exposure deserves further evaluation in larger cohorts.

Consistent with this advanced disease stage, the cohort also demonstrated a substantial systemic inflammatory and nutritional burden at the time of surgery, with elevated inflammatory markers, anemia, and hypoalbuminemia frequently observed. These findings reflect the chronic inflammatory state and metabolic depletion commonly associated with refractory CD and underline the importance of multidisciplinary perioperative management in this population [29,30,31].

Overall, the cohort predominantly consisted of patients with ileal or ileocolonic Crohn’s disease exhibiting complicated behavior (stricturing or penetrating), most of whom had already been exposed to biologic therapy prior to surgery. This profile reflects a population with biologically active, structurally advanced and treatment-refractory Crohn’s disease, providing clinical context for the need for surgical intervention observed in this study.

### 4.2. Determinants of Surgical Approach and Intraoperative Management

In our cohort, the choice between a laparoscopic and an open surgical approach represents an important decision point in managing complex Crohn’s disease (CD). Although minimally invasive techniques are generally preferred for intestinal resections, their feasibility largely depends on the biological and anatomical severity of the disease at the time of surgery. These observations are consistent with the systematic review and meta-analysis by Cassaro et al., who reported shorter hospital stays and fewer minor postoperative complications following laparoscopic surgery when the minimally invasive approach is feasible [32]. Although conducted in a pediatric and young adult population, these findings support the broader surgical principle favoring minimally invasive approaches when anatomical conditions allow. In our cohort, slightly more than half of the procedures were completed laparoscopically, while a substantial proportion required laparotomy. Rather than reflecting technical limitations, this distribution likely mirrors the advanced disease burden typically encountered in specialized centers, where patients often present with complicated phenotypes and have frequently reached the limits of medical therapy [2,23,28]. To better understand the factors influencing the operative strategy in our cohort, we further examined the clinical and biological determinants associated with the choice of surgical approach.

Markers of systemic disease severity were important determinants of the operative strategy in our cohort. In particular, preoperative hypoalbuminemia emerged as an independent determinant associated with the need for an open surgical approach (*p* = 0.033). Serum albumin is widely recognized as a marker reflecting both systemic inflammation and nutritional depletion in Crohn’s disease, conditions associated with impaired wound healing and increased risk of septic complications after intestinal surgery. Several studies have similarly shown that preoperative hypoalbuminemia predicts postoperative morbidity and intra-abdominal septic complications following Crohn’s disease resections as well as other types of surgical interventions [16,33,34]. These findings support the concept that albumin reflects the overall inflammatory and nutritional burden of the disease and highlight the importance of careful perioperative optimization before intestinal surgery, as emphasized in perioperative management studies in inflammatory bowel disease. In such cases, preoperative optimization, including “prehabilitation” strategies aimed at improving nutritional reserves and controlling inflammatory burden prior to surgery, may contribute to safer operative conditions and improved postoperative recovery [29,30,31].

Local septic complications also influenced operative strategy. In our cohort, intra-abdominal abscesses were independently associated with the need for an open surgical approach in multivariate analysis. Sepsis may distort anatomical planes and create dense inflammatory adhesions, making minimally invasive dissection technically challenging and increasing operative complexity. Similar associations between intra-abdominal sepsis and difficult surgical conditions have been reported in previous Crohn’s disease surgical series [23,27].

A comparable pattern was observed in patients with penetrating disease behavior. Although intra-abdominal fistulas and the B3 phenotype did not remain independent predictors in multivariate analysis, both showed a clear tendency toward open surgery in our cohort. Penetrating Crohn’s disease is frequently associated with inflammatory masses, fistulous tracts, and distorted anatomy, factors widely recognized as drivers of surgical complexity. This is consistent with the observed trend toward laparotomy in patients with fistulizing disease in our cohort. In addition, postoperative healing in patients with fistulizing Crohn’s disease is known to be particularly challenging due to the extensive inflammatory tissue damage and distorted anatomy associated with penetrating disease [5,35].

Urgent interventions also tended to require laparotomy more frequently than elective procedures, reflecting the clinical context of acute complications such as bowel obstruction or uncontrolled intra-abdominal sepsis, where rapid access and reliable source control may take precedence over minimally invasive techniques. Conversely, previous abdominal surgeries did not significantly influence the surgical approach in our cohort, suggesting that current inflammatory burden and anatomical complexity may be more relevant determinants of operative feasibility than prior operative scars alone [2].

Surgical intervention frequently required technically demanding procedures, with 60.0% of patients (*n* = 36) necessitating combined resection procedures, defined in our analysis as intestinal resections associated with additional intraoperative maneuvers such as stoma formation, intra-abdominal abscess drainage, or stricturoplasty. This high procedural burden likely reflects the referral pattern typical of a tertiary center, where patients often present with advanced structural disease requiring more extensive intraoperative management [2]. In such specialized environments, surgery is often prioritized for patients with extensive structural damage who have already reached the “therapeutic ceiling” of medical therapy, often after prolonged disease evolution and exposure to multiple lines of biologic therapy, resulting in a high overall disease burden and limited remaining medical options at the time of surgical referral [36].

To identify the primary drivers of this increased intraoperative burden, we performed a comprehensive univariate screening followed by multivariate logistic regression. Our univariate analysis identified several clinical and biological factors associated with the need for combined resection procedures (Supplementary Table S2). The strongest anatomical associations were observed for penetrating disease features: intra-abdominal abscesses were found exclusively in this group (63.9% vs. 0%, *p* < 0.001), while intra-abdominal fistulas were significantly more prevalent (52.8% vs. 4.2%, *p* < 0.001) [23]. The exclusive association between intra-abdominal abscesses and combined resection procedures further highlights the central role of local sepsis in driving the need for more extensive intraoperative management [37]. Clinically and biologically, these patients presented with a significantly higher inflammatory and nutritional burden, including preoperative hypoalbuminemia (*p* = 0.031) and markedly elevated CRP levels (>50 mg/L, *p* = 0.001) [16,33,38]. However, in multivariate analysis, only anatomical factors, particularly intra-abdominal fistulas, remained independently associated with combined resection procedures, while inflammatory and nutritional markers lost statistical significance. This suggests that structural disease severity is a more accurate determinant of procedural complexity, outweighing purely laboratory-based markers in the final operative decision [33,39]. This advanced disease burden is often compounded by the patient’s overall condition; as noted by Cassaro et al. (2025), IBD patients may present at surgery in a physically and nutritionally compromised state, which may further impair tissue integrity and increase the overall surgical burden [32].

Furthermore, our analysis identified a critical clinical link between intraoperative burden and the subsequent medical strategy. The requirement of combined resection procedures was independently associated with a higher rate of postoperative biologic intensification (OR = 5.56, 95% CI: 1.05–29.57, *p* = 0.044). In a cohort where 21.7% of patients had already failed multiple biologic lines, this finding reflects an association between intraoperative disease burden and postoperative biologic intensification and should be interpreted cautiously, without implying a causal relationship or a direct indication for therapeutic escalation [1,2,19,36].

Taken together, these findings suggest that surgical management in Crohn’s disease is primarily driven by the biological severity and structural complications of the disease rather than by purely technical considerations. In this context, advanced structural damage, particularly penetrating disease and intra-abdominal sepsis, frequently necessitates more extensive intraoperative management and may limit the feasibility of minimally invasive approaches. While laparoscopy remains the preferred strategy whenever feasible, severe nutritional depletion and septic complications often require an open approach to ensure safe management of complex inflammatory lesions. These observations further support the role of surgery as a complementary strategy in patients with limited remaining therapeutic options.

### 4.3. Drivers of Fecal Diversion

The utilization of fecal diversion (stoma) was a central component of risk management in our cohort, required in 43.3% (*n* = 26) of cases. This high rate reflects the role of staged surgical strategies in a refractory cohort, where a temporary stoma is often used to ensure safety in high-risk operative environments. Our data suggest a distinct divergence in the clinical drivers dictating the type of diversion, indicating that the surgical strategy is governed primarily by the underlying disease phenotype rather than by the medical therapy administered at the time of surgery [2,40].

The role of this phenotypic driver was first suggested in our univariate screening, where distinct patterns emerged between the two types of diversion (Supplementary Table S3). Ileostomy formation was primarily associated with intra-abdominal fistulizing disease (*p* = 0.034), suggesting that diversion in these cases reflects the consequences of the penetrating abdominal phenotype and the need to protect anastomoses in a hostile intra-abdominal environment. Penetrating Crohn’s disease is known to significantly alter the operative field through inflammatory masses, fistulas, and localized sepsis, frequently necessitating diversion in order to safely avoid diseased intestinal segments and protect anastomoses [5,23,41].

Colostomy formation was associated with a broader set of disease severity markers identified in the univariate analysis, including intra-abdominal fistulas (*p* = 0.013), abscesses (*p* = 0.045), preoperative hypoalbuminemia (*p* = 0.022), and left-sided colonic disease requiring hemicolectomy (*p* < 0.001). These findings suggest that diversion in this setting likely reflects the combined impact of local septic complications, anatomical complexity, and systemic disease burden. In the multivariate analysis, perianal disease remained independently associated with colostomy formation (OR = 12.199, *p* = 0.014), while the other variables did not retain statistical significance after adjustment, likely reflecting their overlap within a broader severe disease phenotype. Overall, this pattern is consistent with established surgical principles in Crohn’s disease, where fecal diversion is frequently required in the presence of compromised tissue integrity and complex disease involvement [30,40,42]. Given the heterogeneity of perianal Crohn’s disease, its specific contribution to diversion strategies may not be fully captured in this analysis and warrants further evaluation in dedicated studies.

An additional observation in our cohort was that preoperative anti-TNF exposure was not associated with an increased likelihood of fecal diversion, regardless of the diversion type (colostomy *p* = 0.264; ileostomy *p* = 1.000). Rather than reflecting the impact of a specific biologic class, this finding supports the concept that surgical decision-making in Crohn’s disease may be primarily driven by the cumulative structural burden of disease, such as fistulas, abscesses, and severe inflammatory damage, rather than by the medical therapy administered at the time of surgery. This interpretation is consistent with contemporary perioperative data suggesting that biologic exposure alone does not independently determine surgical outcomes, while the anatomical severity of disease remains a key determinant of operative strategy [2,18,29,43].

Overall, these findings suggest that fecal diversion strategies reflect different manifestations of disease severity in Crohn’s disease. Both colostomy and ileostomy were associated with markers of advanced disease, including intra-abdominal fistulas, abscesses, and hypoalbuminemia, although with partially distinct patterns, with colostomy more frequently linked to distal disease involvement, and ileostomy to penetrating intra-abdominal disease. In this context, diversion should not be interpreted solely as a failure of medical therapy, but rather as a surgical response to structural complications of the disease [2,41,44].

### 4.4. Surgical Burden and Postoperative Outcomes

Postoperative outcomes in this cohort were characterized by a relatively low rate of severe complications despite the underlying disease complexity. Early adverse events were predominantly infectious and only rarely required surgical reintervention, indicating an overall acceptable immediate postoperative course. No meaningful differences in complication rates were observed according to preoperative anti-TNF exposure, suggesting no significant association between perioperative biological therapy and short-term surgical safety in this cohort.

In contrast, the longitudinal surgical course revealed that the observed cumulative surgical burden reflects two distinct processes: disease progression and staged surgical management. Although nearly half of the patients required additional interventions during follow-up, most reinterventions consisted of planned reconstructive procedures, particularly stoma reversal, rather than true disease-related failure. Temporary fecal diversion is frequently used in Crohn’s disease to control intra-abdominal sepsis or protect high-risk anastomoses before definitive reconstruction is attempted. In such cases, subsequent stoma reversal represents a planned step within the overall surgical management of the disease. Similar observations have been reported in previous studies emphasizing that surgical management of Crohn’s disease often involves staged strategies, particularly in patients with penetrating or complicated phenotypes [25,41].

A smaller subset of patients required additional surgery due to an unfavorable disease course, including further resections or reoperations performed outside the immediate postoperative setting, reflecting persistent or progressive structural disease rather than acute surgical failure. Some patients required multiple subsequent procedures, consistent with the chronic and relapsing nature of Crohn’s disease. When both prior and post-index interventions are considered, a substantial proportion of patients required multiple abdominal surgeries, highlighting the cumulative surgical burden associated with long-standing disease [45].

In this context, cumulative surgical burden should be interpreted with caution, as not all reinterventions reflect disease recurrence. While early postoperative outcomes were acceptable, the longitudinal course reflects the complex and staged nature of surgical management in Crohn’s disease. In complex cases, particularly in tertiary referral settings, surgical management often follows a staged approach in which temporary fecal diversion and subsequent restoration of bowel continuity represent planned steps aimed at achieving source control while preserving long-term intestinal function, rather than indicators of unfavorable disease evolution [21,26].

### 4.5. Postoperative Therapeutic Strategy and Biological Outcomes

Postoperative medical management in our cohort reflected the substantial therapeutic demand typical of patients with advanced and treatment-refractory Crohn’s disease. In our study, biologic intensification was defined as either the initiation of a first-line biologic agent in previously naive patients or switching to a different biologic class after surgery. Using this definition, biologic intensification was required in approximately one third of patients at discharge, while nearly half of the cohort continued the same biologic therapy they had received preoperatively (Supplementary Table S4). This distribution illustrates the heterogeneous therapeutic context in which surgery was performed, ranging from biologic-naive individuals to patients with extensive prior exposure to advanced therapies.

When we explored the clinical drivers of this postoperative strategy, combined resection procedures were associated with a higher rate of biologic intensification (OR 5.56, 95% CI: 1.05–29.57, *p* = 0.044). From a clinical perspective, this association may reflect the fact that technically demanding procedures identify a subgroup of patients with a particularly aggressive disease trajectory who require closer therapeutic control after surgery. Although some clinicians prefer to maintain the same biologic agent after surgery, biologic optimization may still become necessary in patients with persistent or high-risk disease features [1,2,44]. In this context, postoperative biologic intensification should not be interpreted as a preoperative predictor of surgical burden, but rather as a marker of postoperative disease severity and therapeutic demand. This may reflect treatment escalation in patients undergoing more extensive or complicated surgical procedures, rather than a causal relationship.

Longitudinal follow-up data further illustrated the ongoing therapeutic demand in this cohort. Over time, an increasing proportion of patients required escalation to second-line or higher biologic therapies, indicating that postoperative management frequently involved continued optimization of immunosuppressive treatment. These findings are consistent with the current understanding that surgery should not be interpreted as the endpoint of medical therapy in Crohn’s disease, but rather as part of an integrated treatment pathway in which surgical resection reduces inflammatory burden and facilitates subsequent medical disease control [1,2,46,47].

The relationship between preoperative biologic exposure and surgical outcomes could not be clearly established in this cohort, likely reflecting the heterogeneity of treatment pathways and the limited sample size.

An additional observation in our cohort was the biological recovery observed after surgery, defined as the simultaneous normalization of hemoglobin, serum albumin, and C-reactive protein, reflecting improvements in systemic inflammation and nutritional status following bowel resection; however, this concept should be interpreted as exploratory rather than a validated clinical endpoint. While this definition integrates parameters reflecting distinct physiological domains, each component is supported by the existing literature, including the use of C-reactive protein as a marker of biological remission, the association of serum albumin with postoperative risk stratification, and the link between anemia and disease activity [11,48,49]. In line with this, combinations of inflammatory and nutritional parameters, including CRP, albumin, and hemoglobin, have been previously explored as composite predictors of postoperative outcomes in Crohn’s disease, in the preoperative setting, supporting the conceptual rationale of the present definition [50]. Similar observations in the surgical literature suggest that removal of severely inflamed bowel segments may reduce systemic inflammatory burden and allow patients with advanced Crohn’s disease to recover from a state of chronic inflammatory stress [46,47,51,52].

In our cohort, this postoperative biological recovery was reflected by improvements in inflammatory and nutritional markers, together with a trend toward decreased fecal calprotectin levels during follow-up in the subset of patients with available data. However, no clinical or therapeutic variables were significantly associated with the achievement of this endpoint, suggesting that this pattern may be primarily driven by the removal of macroscopically diseased bowel rather than identifiable predictors. This observation may also be influenced by the limited sample size, which restricts the ability to detect significant associations. Although no statistically significant predictors were identified, these findings provide a descriptive perspective on postoperative recovery dynamics in patients with advanced Crohn’s disease undergoing surgery. We conceptualized this pattern as a postoperative biological response, although it should be interpreted as an exploratory observation rather than a validated clinical endpoint [43].

Endoscopic reassessment, although limited by the retrospective design and substantial missingness for SES-CD and Rutgeerts scores, showed encouraging results in the evaluated subset, with a proportion of patients achieving endoscopic remission and low-risk postoperative recurrence patterns. As a result, these findings should be interpreted with caution and considered supportive but not definitive evidence for the overall study conclusions.

Despite overall stabilization, a subset of patients continued to exhibit biochemical evidence of low-grade inflammatory activity during follow-up. This highlights an important clinical nuance: surgery may provide physiological stabilization rather than definitive disease resolution. These findings suggest that surgical removal of diseased bowel segments may be associated with improvements in biological parameters, potentially creating a window of opportunity for postoperative stabilization.

The rate of complete postoperative biological normalization, defined as the simultaneous normalization of hemoglobin, albumin, and CRP, was relatively low. However, this composite endpoint integrates parameters reflecting distinct physiological domains, including inflammation, nutritional status, and anemia, and should therefore be interpreted with caution.

Importantly, when analyzed individually, these parameters demonstrated consistent improvement following surgery, with high rates of hemoglobin and albumin normalization and a marked reduction in inflammatory markers. These findings suggest that the composite endpoint may underestimate the overall clinical benefit of surgical intervention, particularly in a cohort characterized by advanced and refractory disease.

Emerging approaches based on artificial intelligence and machine learning may enhance the prediction of surgical outcomes and support individualized management strategies in Crohn’s disease. By integrating multidimensional clinical, biological, and imaging data, these models may improve risk stratification in complex patients. However, their role in routine clinical practice remains to be further validated [53].

### 4.6. Strengths and Limitations

#### 4.6.1. Strengths

This study has several strengths. First, it provides a detailed surgical characterization of Crohn’s disease patients treated in a tertiary referral center, a setting where complex and refractory cases are commonly managed. Second, the study integrates clinical, surgical, and biological parameters, allowing a comprehensive evaluation of postoperative recovery beyond purely technical surgical outcomes. Third, the use of composite endpoints such as combined resection procedures and the exploratory concept of postoperative biological response allowed us to explore operative burden and postoperative biological recovery in a structured manner, providing additional insight into the interaction between surgical intervention and disease control.

#### 4.6.2. Limitations

This study also has several limitations that should be considered when interpreting the findings. First, its retrospective design and the relatively small cohort size (*N* = 60) from a single gastroenterology unit within a specialized referral center may limit the applicability of the results to broader patient populations and reduce the statistical power of the multivariable analyses. In addition, the specialized referral setting likely enriched the cohort with patients presenting more advanced or refractory disease, which may further limit the applicability of the findings to the broader Crohn’s disease population. Furthermore, the extended inclusion period may have introduced temporal heterogeneity, as both biologic therapies and surgical strategies have evolved over time.

Second, postoperative endoscopic follow-up was available only for a subset of patients. SES-CD reassessment at six months was performed in approximately 35% of the cohort, while Rutgeerts scores were available in only 16.7% of cases. Preoperative SES-CD evaluation was also unavailable for some patients. Similarly, fecal calprotectin measurements were missing in a substantial proportion of cases, partly reflecting the limited reimbursement of this biomarker in the Romanian healthcare system during the study period. Additionally, long-term follow-up data were not uniformly available, as a proportion of patients continued postoperative care in other centers, which may have contributed to incomplete outcome capture. These limitations restricted the ability to perform more detailed analyses of endoscopic and biomarker-based outcomes.

Third, the study used cohort-specific composite definitions for combined resection procedures and the exploratory concept of postoperative biological response. Although these definitions were clinically motivated and intended to better reflect the operative burden of advanced Crohn’s disease, they have not yet been externally validated and should therefore be interpreted with caution.

Finally, postoperative medical management was not standardized and reflected real-world clinical practice, which may have introduced heterogeneity in treatment strategies. In addition, despite comprehensive institutional records, it remains possible that some reinterventions performed in other centers were not fully captured during follow-up.

The multivariate model for combined resection procedures was constructed using a limited number of variables to reduce the risk of overfitting, given the relatively small sample size. Variable selection was based on both statistical significance in univariate analysis and clinical relevance. Despite these precautions, the results of the multivariable analysis should be interpreted with caution due to the limited number of events, which can lead to overfitting and lack of generalizability, as reflected by the wide confidence intervals observed in some estimates. Therefore, these findings should be considered primarily exploratory and hypothesis-generating.

Despite these limitations, the study provides clinically relevant insights into surgical decision-making and postoperative management in a real-world cohort of patients with complex Crohn’s disease treated in a specialized referral center.

## 5. Conclusions

In this real-world cohort of patients with complex Crohn’s disease treated in a tertiary referral center, surgery was predominantly required for individuals with advanced structural disease, often reflecting a therapeutic ceiling of medical therapy. Structural disease severity, particularly intra-abdominal fistulas and the perianal disease pattern, emerged as key determinants of operative burden and the need for fecal diversion, highlighting the central role of disease phenotype in surgical decision-making. Additional factors such as intra-abdominal abscesses and hypoalbuminemia were associated with increased operative burden.

Despite the substantial operative burden observed in this cohort, surgical intervention was associated with improvements in inflammatory and nutritional markers following effective source control, although complete postoperative biological normalization was achieved in a limited proportion of patients. These findings support the concept that surgery in Crohn’s disease should not be interpreted solely as a failure of medical therapy, but rather as an integral component of multidisciplinary disease management.

Surgical intervention may provide a window of physiological stabilization that facilitates subsequent medical optimization. Future multidisciplinary strategies may benefit from the identification of high-risk phenotypic markers to better characterize this therapeutic window and help refine therapeutic strategies in Crohn’s disease patients, including disease phenotypes such as perianal involvement, which was not the primary focus of the present analysis and warrants further dedicated investigation.

## Figures and Tables

**Table 1:** Baseline demographic and disease phenotype characteristics of the study cohort (*N* = 60).

Variable	Category	*n* (%) or Median (IQR)
**Age (years)**	Age at diagnosis	28 (21.0–33.8)
	Age at index surgery	32.5 (25.3–41.0)
**Sex**	Male	34 (56.7%; 95% CI: 44.1–69.3)
	Female	26 (43.3%; 95% CI: 30.7–55.9)
**Environment**	Urban	47 (78.3%; 95% CI: 67.9–88.7)
	Rural	13 (21.7%; 95% CI: 11.3–32.1)
**Smoking status**	Active smoker	19 (31.7%; 95% CI: 19.9–43.5)
	Non-smoker	41 (68.3%; 95% CI: 56.5–80.1)
**Montreal A (Age at onset)**	A1 (≤16 years)	3 (5.0%; 95% CI: 0–10.5)
	A2 (17–39 years)	47 (78.3%; 95% CI: 67.9–88.7)
	A3 (≥40 years)	10 (16.7%; 95% CI: 7.3–26.1)
**Montreal L (Location)**	L1 (Ileal)	22 (36.7%; 95% CI: 24.5–48.9)
	L2 (Colonic)	12 (20.0%; 95% CI: 9.9–30.1)
	L3 (Ileocolonic)	26 (43.3%; 95% CI: 30.7–55.9)
	L4 (Upper GI modifier)	4 (6.7%; 95% CI: 0.4–13.0)
**Montreal B (Behavior)**	B1 (Inflammatory)	2 (3.3%; 95% CI: 0–7.9)
	B2 (Stricturing)	34 (56.7%; 95% CI: 44.1–69.3)
	B3 (Penetrating)	24 (40.0%; 95% CI: 27.6–52.4)
	Perianal modifier (p)	12 (20.0%; 95% CI: 9.9–30.1)
**Surgical history**	Prior surgery (abdominal or perianal)	27 (45.0%; 95% CI: 32.4–57.6)
	1 prior procedure	17 (28.3%; 95% CI: 16.9–39.7)
	2 prior procedures	9 (15.0%; 95% CI: 6.0–24.0)
	3 prior procedures	1 (1.7%; 95% CI: 0–5.0)
	Prior appendectomy	15 (25.0%; 95% CI: 14.0–36.0)
**Comorbidities**	Overall presence	18 (30.0%; 95% CI: 18.4–41.6)
	Rheumatologic manifestations	5 (8.3%; 95% CI: 1.3–15.3)
	Cardiovascular comorbidities	4 (6.7%; 95% CI: 0.4–13.0)
	Ankylosing spondylitis	4 (6.7%; 95% CI: 0.4–13.0)
	Pyoderma gangrenosum	2 (3.3%; 95% CI: 0–7.9)

Note: GI = gastrointestinal; *n* = number. Montreal classification: A = age at diagnosis, L = disease location, B = disease behavior; p = perianal disease modifier. Data are presented as *n* (%) or median (IQR). Only the most frequent comorbidities are listed; categories are not mutually exclusive, and some patients may present multiple comorbidities. L4 represents an additional modifier and may overlap with L1–L3 categories.

**Table 2:** Preoperative clinical, laboratory, and therapeutic characteristics of the study cohort (*N* = 60).

Variable	Category	*n* (%) or Median (IQR)
**Laboratory profile**	Hemoglobin (g/dL)	12.1 (10.6–13.6)
	Anemia (overall)	23 (39.7%; 95% CI: 27.1–52.3)
	Mild (10–12 g/dL)	14 (24.1%; 95% CI: 13.1–35.1)
	Moderate/Severe (<10 g/dL)	9 (15.5%; 95% CI: 6.2–24.8)
	Serum albumin (g/dL)	4.0 (3.3–4.6)
	Hypoalbuminemia (<3.5 g/dL)	15 (25.9%; 95% CI: 14.6–37.2)
	CRP (mg/L)	37.0 (5.5–92.0)
	CRP elevated (>5 mg/L)	33 (56.9%; 95% CI: 44.1–69.7)
	Fibrinogen elevated (>400 mg/dL)	35 (60.3%; 95% CI: 47.7–72.9)
	Ferritin (ng/mL) (*n* = 17)	177 (72.5–469.0)
	Fecal calprotectin (µg/g) (*n* = 16)	820 (284.3–1675.0)
**Objective evaluation**	Preoperative imaging (CT/MRI/US)	57 (95.0%; 95% CI: 89.5–100)
	Endoscopic activity (SES-CD) (*n* = 40)	
	Remission (0–2)	2 (5.0%; 95% CI: 0–11.8)
	Mild activity (3–6)	13 (32.5%; 95% CI: 18.0–47.0)
	Moderate activity (7–15)	19 (47.5%; 95% CI: 32.0–63.0)
	Severe activity (≥16)	6 (15.0%; 95% CI: 3.9–26.1)
**Medical therapy (last 6 months)**	Any IBD treatment	45 (75.0%; 95% CI: 63.9–86.1)
**Conventional therapy**	Corticosteroids	9 (15.0%; 95% CI: 6.0–24.0)
	5-Aminosalicylates	2 (3.3%; 95% CI: 0–7.9)
	Immunomodulators (Azathioprine)	15 (25.0%; 95% CI: 14.0–36.0)
**Advanced therapy exposure**	Total biologic/targeted therapy	41 (68.3%; 95% CI: 56.5–80.1)
**Anti-TNF agents**	Anti-TNF subtotal	28 (46.7%; 95% CI: 33.9–59.5)
	Adalimumab	16 (26.7%; 95% CI: 15.5–37.9)
	Infliximab	12 (20.0%; 95% CI: 9.9–30.1)
**Newer biologics**	Newer biologics subtotal	13 (21.7%; 95% CI: 11.3–32.1)
	Ustekinumab	9 (15.0%; 95% CI: 6.0–24.0)
	Vedolizumab	4 (6.7%; 95% CI: 0.4–13.0)
**Number of therapeutic lines**	0 lines (biologic-naive)	19 (31.7%; 95% CI: 19.9–43.5)
	1 line	28 (46.7%; 95% CI: 33.9–59.5)
	2 lines	10 (16.7%; 95% CI: 7.3–26.1)
	3 lines	2 (3.3%; 95% CI: 0–7.9)
	4 lines	1 (1.7%; 95% CI: 0–5.0)

Note: CRP = C-reactive protein; SES-CD = Simple Endoscopic Score for Crohn’s Disease; *n* = number. Data are presented as *n* (%) or median (IQR). Laboratory values were available for 58 patients; percentages are calculated based on available cases. Endoscopic activity (SES-CD) was available for 40 patients. Ferritin measurements were available for 17 patients. Fecal calprotectin measurements were available for 16 patients because the test is not routinely reimbursed within the Romanian healthcare system.

**Table 3:** Operative characteristics (*N* = 60).

Variable	Category	*n* (%; 95% CI)
**Operative setting**	Elective	44 (73.3%; 95% CI: 62.1–84.5)
	Urgent	16 (26.7%; 95% CI: 15.5–37.9)
**Surgical approach**	Laparoscopic	32 (53.3%; 95% CI: 40.7–65.9)
	Laparotomy (open)	28 (46.7%; 95% CI: 34.1–59.3)
**Resection type**	Resection with primary anastomosis	34 (56.7%; 95% CI: 44.1–69.3)
	Resection with stoma	26 (43.3%; 95% CI: 30.7–55.9)
	Ileostomy	18 (30.0%; 95% CI: 18.4–41.6)
	Colostomy	8 (13.3%; 95% CI: 4.7–21.9)

Note: Ileostomy and colostomy are subcategories of resection with stoma; percentages are calculated based on the total cohort (*N* = 60).

**Table 4:** Univariate and multivariate analysis of factors associated with the surgical approach (*N* = 60).

Variable	Laparoscopy (*n* = 32)	Laparotomy (*n* = 28)	Univariate *p*-Value	Multivariate OR (95% CI)	*p*-Value
**Surgical Urgency (Urgent)**	5 (15.6%)	11 (39.3%)	**0.047**	3.11 (0.84–11.51)	0.090
**Abscess/Collection**	8 (25.0%)	15 (53.6%)	**0.034**	**3.46 (1.06–11.37)**	**0.040**
**Preoperative Hypoalbuminemia**	4 (13.3%)	11 (39.3%)	**0.036**	**4.46 (1.13–17.67)**	**0.033**
**Intra-abdominal Fistulas**	7 (21.9%)	13 (46.4%)	0.058	–	–
**Penetrating Behavior (B3)**	9 (28.1%)	15 (53.6%)	0.065	–	–
**Elevated CRP (>5 mg/L)**	14 (46.7%)	19 (67.9%)	0.120	–	–
**Previous Abdominal Surgeries**	15 (46.9%)	12 (42.9%)	0.799	–	–
**Multiple Strictures/>5 cm**	15 (46.9%)	10 (35.7%)	0.439	–	–

Note: Significant *p*-values (<0.05) are highlighted in bold. Percentages for laboratory variables were calculated using the number of patients with available laboratory data (*n* = 58). Variables with *p* < 0.1 in univariate analysis and deemed clinically relevant were evaluated for inclusion in the multivariable model. To reduce the risk of overfitting given the limited sample size, the number of variables included in the final model was restricted.

**Table 5:** Multivariate logistic regression for factors associated with combined resection procedures (*N* = 60).

Variable	OR (Exp(B))	95% C.I. for OR	*p*-Value
**Intra-abdominal Fistulas**	22.93	2.40–219.37	**0.007**
**Biologic Intensification**	5.56	1.05–29.57	**0.044**
**Preoperative Elevated CRP**	3.55	0.66–19.21	0.141
**Preoperative Hypoalbuminemia**	2.67	0.32–22.58	0.367

Note: Significant *p*-values (<0.05) are highlighted in bold. Biologic intensification is a postoperative variable and was included for exploratory purposes; it should not be interpreted as a predictor of intraoperative surgical decisions.

**Table 6:** Multivariate logistic regression for independent predictors of colostomy formation (*N* = 60).

Variable	B	S.E.	Wald	df	*p*-Value	Odds Ratio (Exp(B))	95% C.I. for OR
**Perianal Pattern**	2.501	1.020	6.016	1	**0.014**	**12.199**	**1.653–90.031**
**Intra-abdominal Fistulas**	1.549	1.015	2.326	1	0.127	4.705	0.643–34.426
**Preoperative Hypoalbuminemia**	1.270	1.025	1.537	1	0.215	3.562	0.478–26.535

Note: B = regression coefficient; S.E. = standard error; Wald = Wald chi-square statistic; df = degrees of freedom; OR = odds ratio; C.I. = confidence interval. The multivariate model was adjusted for variables with *p* < 0.1 in the univariate analysis (perianal pattern, intra-abdominal fistulas, and preoperative hypoalbuminemia). The perianal disease pattern was the only independent factor associated with colostomy formation (OR = 12.199, *p* = 0.014).

**Table 7:** Postoperative Complications and Management Profile (*N* = 60).

Category	Outcome	*n* (%; 95% CI)
**Timing**	Early (≤30 days)	12 (20.0%; 95% CI: 9.9–30.1)
	Late (>30 days)	2 (3.3%; 95% CI: 0–7.9)
**Specific Complications**	Postoperative infectious complications (non-CDI)	6 (10.0%; 95% CI: 2.4–17.6)
	Clostridioides difficile infection (CDI)	2 (3.3%; 95% CI: 0–7.9)
	Acute kidney injury (AKI)	1 (1.7%; 95% CI: 0–5.0)
	Anastomotic dehiscence	1 (1.7%; 95% CI: 0–5.0)
	Postoperative hemorrhage	1 (1.7%; 95% CI: 0–5.0)
	Evisceration	1 (1.7%; 95% CI: 0–5.0)
	Postoperative ileus	2 (3.3%; 95% CI: 0–7.9)
	Thrombosis (late)	1 (1.7%; 95% CI: 0–5.0)
	Postoperative stenosis (late, endoscopically managed)	1 (1.7%; 95% CI: 0–5.0)
**Management**	Non-surgical management	11 (18.3%; 95% CI: 8.5–28.1)
	Surgical reintervention (≤30 days)	3 (5.0%; 95% CI: 0–10.5)

Note: Data are presented as *n* (%; 95% CI), calculated relative to the total cohort (*N* = 60). Some patients experienced more than one postoperative complication (e.g., prolonged ileus occurring in association with infectious complications); therefore, the total number of complication events exceeds the number of affected patients.

**Table 8:** Postoperative Biologic Management and Longitudinal Therapeutic Evolution (*N* = 60).

Period/Strategy Category	Therapeutic Status	*n* (%; 95% CI)
**Initial Postoperative Strategy**		
**Biologic intensification**	Initiation of first-line biologic	14 (23.3%; 95% CI: 12.6–34.0)
	Switch to a different biologic agent	7 (11.7%; 95% CI: 3.6–19.8)
	**Subtotal intensification**	21 (35.0%; 95% CI: 22.9–47.1)
**Non-intensification**	Maintenance of preoperative biologic	29 (48.3%; 95% CI: 35.7–60.9)
	No postoperative biologic therapy	2 (3.3%; 95% CI: 0–7.9)
	**Subtotal non-intensification**	31 (51.7%; 95% CI: 39.1–64.3)
**Unknown**	Strategy unavailable at discharge	8 (13.3%; 95% CI: 4.7–21.9)
**Six Months Postoperative**		
	Remaining on first-line biologic	30 (50.0%; 95% CI: 37.4–62.6)
	Second-line biologic therapy	13 (21.7%; 95% CI: 11.3–32.1)
	Third- or fourth-line biologic therapy	4 (6.7%; 95% CI: 0.4–13.0)
	Unknown	13 (21.7%; 95% CI: 11.3–32.1)
	Introduction of an additional biologic agent during follow-up *	10 (16.7%; 95% CI: 7.3–26.1)
**Twelve Months Postoperative**		
	Remaining on first-line biologic	26 (43.3%; 95% CI: 30.7–55.9)
	Second-line biologic therapy	17 (28.3%; 95% CI: 16.9–39.7)
	Third- or fourth-line biologic therapy	4 (6.7%; 95% CI: 0.4–13.0)
	Unknown	13 (21.7%; 95% CI: 11.3–32.1)

Note: * Introduction of an additional biologic agent during follow-up represents patients in whom a second biologic agent was introduced postoperatively. Percentages are calculated based on the total cohort of 60 patients.

## Data Availability

The data presented in this study are available from the corresponding author upon reasonable request and subject to institutional and ethical restrictions.

## Supplementary Materials

The following supporting information can be downloaded at: https://www.mdpi.com/article/10.3390/medicina62050917/s1, Table S1. Anatomical disease features and surgical procedures (N = 60); Table S2. Univariate analysis of factors associated with combined resection procedures (N = 60); Table S3. Univariate analysis of predictors associated with ileostomy and colostomy (N = 60); Table S4. Correlation between Clinical and Therapeutic Factors and the Achievement of Postoperative Biological Response at 6 Months; Table S5. Cumulative Surgical Burden and Longitudinal Evolution (N = 60).

## Abbreviations

The following abbreviations are used in this manuscript:

AKI	Acute kidney injury
CD	Crohn’s disease
CDI	Clostridioides difficile infection
CRP	C-reactive protein
CT	Computed tomography
GI	Gastrointestinal
IBD	Inflammatory bowel disease
ICD	International Classification of Diseases
IQR	Interquartile range
MRI	Magnetic resonance imaging
OR	Odds ratio
SES-CD	Simple Endoscopic Score for Crohn’s Disease
SPSS	Statistical Package for the Social Sciences
TNF	Tumor necrosis factor

## References

[1] Gordon H., Minozzi S., Kopylov U., Verstockt B., Chaparro M., Buskens C., Warusavitarne J., Agrawal M., Allocca M., Atreya R. (2024). ECCO Guidelines on Therapeutics in Crohn’s Disease: Medical Treatment. J. Crohns Colitis.

[2] Adamina M., Minozzi S., Warusavitarne J., Buskens C.J., Chaparro M., Verstockt B., Kopylov U., Yanai H., Vavricka S.R., Sigall-Boneh R. (2024). ECCO Guidelines on Therapeutics in Crohn’s Disease: Surgical Treatment. J. Crohns Colitis.

[3] Ponsioen C.Y., de Groof E.J., Eshuis E.J., Gardenbroek T.J., Bossuyt P.M.M., Hart A., Warusavitarne J., Buskens C.J., van Bodegraven A.A., Brink M.A. (2017). Laparoscopic ileocaecal resection versus infliximab for terminal ileitis in Crohn’s disease: A randomised controlled, open-label, multicentre trial. Lancet Gastroenterol. Hepatol..

[4] Oldenburg L., Haanappel A.E.G., Ali M., Bosman C., Buskens C.J., Ponsioen C.Y., D’Haens G.R., Bemelman W.A. (2026). Ileocaecal resection versus infliximab for ileal Crohn’s disease: Retrospective 10-year follow-up of the LIR!C trial. Lancet Gastroenterol. Hepatol..

[5] Avellaneda N., Maroli A., Pellino G., Carvello M., Tottrup A., Bislenghi G., Colpaert J., D’Hoore A., Giorgi L., Juachon P. (2025). Long-term comparative outcomes after ileocecal resection for inflammatory versus complicated Crohn’s disease: A multicenter, retrospective study (Crohn’s-Urg). Dig. Liver Dis..

[6] Preda C.M., Istratescu D., Nitescu M., Manuc T., Manuc M., Stroie T., Catrinoiu M., Tieranu C., Meianu C.G., Tugui L. (2023). Impact of dietary patterns in inflammatory bowel disease subtypes versus healthy subjects: A retrospective cohort study. Maedica.

[7] Wang F.T., Lin Y., Yuan X.Q., Gao R.Y., Wu X.C., Xu W.W., Wu T.Q., Xia K., Jiao Y.R., Yin L. (2024). Predicting short-term major postoperative complications in intestinal resection for Crohn’s disease: A machine learning-based study. World J. Gastrointest. Surg..

[8] Feagan B.G., Sandborn W.J., Gasink C., Jacobstein D., Lang Y., Friedman J.R., Blank M.A., Johanns J., Gao L.L., Miao Y. (2016). Ustekinumab as induction and maintenance therapy for Crohn’s disease. N. Engl. J. Med..

[9] Ferrante M., Panaccione R., Baert F., Bossuyt P., Colombel J.F., Danese S., Dubinsky M., Feagan B.G., Hisamatsu T., Lim A. (2022). Risankizumab as maintenance therapy for moderately to severely active Crohn’s disease: Results from the multicentre, randomised, double-blind, placebo-controlled, withdrawal phase 3 FORTIFY maintenance trial. Lancet.

[10] Loftus E.V., Panés J., Lacerda A.P., Peyrin-Biroulet L., D’Haens G., Panaccione R., Reinisch W., Louis E., Chen M., Nakase H. (2023). Upadacitinib induction and maintenance therapy for Crohn’s disease. N. Engl. J. Med..

[11] Turner D., Ricciuto A., Lewis A., D’Amico F., Dhaliwal J., Griffiths A.M., Bettenworth D., Sandborn W.J., Sands B.E., Reinisch W. (2021). STRIDE-II: An update on the Selecting Therapeutic Targets in Inflammatory Bowel Disease (STRIDE) initiative of the International Organization for the Study of IBD (IOIBD): Determining therapeutic goals for treat-to-target strategies in IBD. Gastroenterology.

[12] Lichtenstein G.R., Loftus E.V., Afzali A., Long M.D., Barnes E.L., Isaacs K.L., Ha C.Y. (2025). ACG clinical guideline: Management of Crohn’s disease in adults. Am. J. Gastroenterol..

[13] Kral J., Nakov R., Lanska V., Barberio B., Benech N., Blesl A., Brunet E., Capela T., Derikx L., Dragoni G. (2023). Significant differences in IBD care and education across Europe: Results of the pan-European VIPER survey. Dig. Dis..

[14] Prokopič M., Gilca-Blanariux G., Lietava P., Trifan A., Pietrzak A., Ladic A., Brinar M., Turcan S., Molnár T., Bánovčin P. (2023). Barriers in inflammatory bowel disease care in Central and Eastern Europe: A region-specific analysis. Ther. Adv. Gastroenterol..

[15] Satsangi J., Silverberg M.S., Vermeire S., Colombel J.F. (2006). The Montreal classification of inflammatory bowel disease: Controversies, consensus, and implications. Gut.

[16] Nguyen G.C., Du L., Chong R.Y., Jackson T.D. (2019). Hypoalbuminaemia and postoperative outcomes in inflammatory bowel disease: The NSQIP surgical cohort. J. Crohns Colitis.

[17] Chen R., Zheng J., Li C., Chen Q., Zeng Z., Li L., Chen M., Zhang S. (2023). Prognostic models for predicting postoperative recurrence in Crohn’s disease: A systematic review and critical appraisal. Front. Immunol..

[18] Schad C.A., Haac B.E., Cross R.K., Syed A., Lonsako S., Bafford A.C. (2019). Early postoperative anti-TNF therapy does not increase complications following abdominal surgery in Crohn’s disease. Dig. Dis. Sci..

[19] Torres J., Mehandru S., Colombel J.F., Peyrin-Biroulet L. (2017). Crohn’s disease. Lancet.

[20] Torres J., Caprioli F., Katsanos K.H., Lobatón T., Micic D., Zerôncio M., Van Assche G., Lee J.C., Lindsay J.O., Rubin D.T. (2016). Predicting outcomes to optimize disease management in inflammatory bowel diseases. J. Crohns Colitis.

[21] Peyrin-Biroulet L., Loftus E.V., Colombel J.F., Sandborn W.J. (2010). The natural history of adult Crohn’s disease in population-based cohorts. Am. J. Gastroenterol..

[22] Mignini I., Blasi V., Termite F., Esposto G., Borriello R., Laterza L., Scaldaferri F., Ainora M.E., Gasbarrini A., Zocco M.A. (2024). Fibrostenosing Crohn’s disease: Pathogenetic mechanisms and new therapeutic horizons. Int. J. Mol. Sci..

[23] Gecse K., Khanna R., Stoker J., Jenkins J.T., Gabe S., Hahnloser D., D’Haens G. (2013). Fistulizing Crohn’s disease: Diagnosis and management. United Eur. Gastroenterol. J..

[24] Rieder F., Fiocchi C., Rogler G. (2017). Mechanisms, management, and treatment of fibrosis in patients with inflammatory bowel diseases. Gastroenterology.

[25] Bernell O., Lapidus A., Hellers G. (2000). Risk factors for surgery and postoperative recurrence in Crohn’s disease. Ann. Surg..

[26] Frolkis A.D., Dykeman J., Negrón M.E., Debruyn J., Jette N., Fiest K.M., Frolkis T., Barkema H.W., Rioux K.P., Panaccione R. (2013). Risk of surgery for inflammatory bowel diseases has decreased over time: A systematic review and meta-analysis of population-based studies. Gastroenterology.

[27] Sakurai Kimura C.M., Scanavini Neto A., Queiroz N.S.F., Horvat N., Camargo M.G.M., Borba M.R., Sobrado C.W., Cecconello I., Nahas S.C. (2021). Abdominal surgery in Crohn’s disease: Risk factors for complications. Inflamm. Intest. Dis..

[28] Bemelman W.A., Warusavitarne J., Sampietro G.M., Serclova Z., Zmora O., Luglio G., de Buck van Overstraeten A., Burke J.P., Buskens C.J., Colombo F. (2018). ECCO-ESCP consensus on surgery for Crohn’s disease. J. Crohns Colitis.

[29] Hazel K., Cooney R. (2025). Preoperative optimization for elective surgery in Crohn’s disease: A narrative review. J. Clin. Med..

[30] Lin C.C., Lin H.H., Chen H.C., Chen N.C., Shih I.L., Hung J.S., Yueh T.C., Chiang F.F., Lin P.W., Tsai Y.Y. (2022). Perioperative optimization of Crohn’s disease. Ann. Gastroenterol. Surg..

[31] Nickerson T.P., Merchea A. (2016). Perioperative considerations in Crohn disease and ulcerative colitis. Clin. Colon Rectal Surg..

[32] Cassaro F., Impellizzeri P., Romeo C., Arena S. (2025). Comparative outcomes of laparoscopic and open surgery in inflammatory bowel disease in pediatric and young adult patients: A systematic review and meta-analysis. J. Gastrointest. Surg..

[33] Ge X., Liu H., Tang S., Wu Y., Pan Y., Liu W., Qi W., Ye L., Cao Q., Zhou W. (2020). Preoperative hypoalbuminemia is an independent risk factor for postoperative complications in Crohn’s disease patients with normal BMI: A cohort study. Int. J. Surg..

[34] Alajmi A., Almehari A., Alzahrani A.R., Aljurays Y., Alzahrani N., Aladel A.M., Alzahrani N. (2024). Impact of preoperative serum albumin level on the outcome of colorectal cancer surgery. Cureus.

[35] Mujukian A., Truong A., Fleshner P., Zaghiyan K. (2020). Long-term healing after complex anal fistula repair in patients with Crohn’s disease. Tech. Coloproctol..

[36] Caron B., Habert A., Bonsack O., Camara H., Jeanbert E., Parigi T.L., Netter P., Danese S., Peyrin-Biroulet L. (2024). Difficult-to-treat inflammatory bowel disease: Effectiveness and safety of 4th and 5th lines of treatment. United Eur. Gastroenterol. J..

[37] de Groof E.J., Carbonnel F., Buskens C.J., Bemelman W.A. (2014). Abdominal abscess in Crohn’s disease: Multidisciplinary management. Dig. Dis..

[38] Solem C.A., Loftus E.V., Tremaine W.J., Harmsen W.S., Zinsmeister A.R., Sandborn W.J. (2005). Correlation of C-reactive protein with clinical, endoscopic, histologic, and radiographic activity in inflammatory bowel disease. Inflamm. Bowel Dis..

[39] Sakurai T., Saruta M. (2023). Positioning and usefulness of biomarkers in inflammatory bowel disease. Digestion.

[40] Hain E., Maggiori L., Orville M., Tréton X., Bouhnik Y., Panis Y. (2019). Diverting stoma for refractory ano-perineal Crohn’s disease: Is it really useful in the anti-TNF era? A multivariate analysis in 74 consecutive patients. J. Crohns Colitis.

[41] Burke J.P. (2019). Role of fecal diversion in complex Crohn’s disease. Clin. Colon Rectal Surg..

[42] Zabot G.P., Cassol O., Saad-Hossne R., Bemelman W. (2020). Modern surgical strategies for perianal Crohn’s disease. World J. Gastroenterol..

[43] Kotze P.G., Ghosh S., Bemelman W.A., Panaccione R. (2017). Preoperative use of anti-tumor necrosis factor therapy in Crohn’s disease: Promises and pitfalls. Intest. Res..

[44] Huynh N., Wang H., Fok K.Y., Toh J.W.T. (2022). What constitutes failure of medical therapy in the changing landscape of Crohn’s disease?. Laparosc. Endosc. Robot. Surg..

[45] Colombo F., Frontali A., Baldi C., Cigognini M., Lamperti G., Manzo C.A., Maconi G., Ardizzone S., Foschi D., Sampietro G.M. (2022). Repeated surgery for recurrent Crohn’s disease: Does the outcome keep worsening operation after operation? A comparative study of 1224 consecutive procedures. Updates Surg..

[46] Chande N., Singh S., Narula N., Gordon M., Kuenzig M.E., Nguyen T.M., MacDonald J.K., Feagan B.G. (2021). Medical management following surgical therapy in inflammatory bowel disease: Evidence from Cochrane reviews. Inflamm. Bowel Dis..

[47] Aljabri R., Al-Saraie S., Alhouti A. (2025). Optimizing biologic therapy for the prevention of post-operative recurrence in Crohn’s disease: Current evidence and future perspectives. Biomedicines.

[48] Evans D.C., Corkins M.R., Malone A., Miller S., Mogensen K.M., Guenter P., Jensen G.L. (2021). The use of visceral proteins as nutrition markers: An ASPEN position paper. Nutr. Clin. Pract..

[49] Bergamaschi G., Castiglione F., D’Incà R., Astegiano M., Fries W., Milla M., Ciacci C., Rizzello F., Saibeni S., Ciccocioppo R. (2023). Prevalence, pathogenesis and management of anemia in inflammatory bowel disease: An IG-IBD multicenter, prospective, and observational study. Inflamm. Bowel Dis..

[50] Ghoneima A.S., Flashman K., Dawe V., Baldwin E., Celentano V. (2019). High risk of septic complications following surgery for Crohn’s disease in patients with preoperative anaemia, hypoalbuminemia and high CRP. Int. J. Color. Dis..

[51] Rathod S., Kumar N., Matiz G.D., Biju S., Girgis P., Sabu N., Mumtaz H., Haider A. (2024). The role of minimally invasive surgery in the management of inflammatory bowel disease: Current trends and future directions. Cureus.

[52] Clough J., Colwill M., Poullis A., Pollok R., Patel K., Honap S. (2024). Biomarkers in inflammatory bowel disease: A practical guide. Ther. Adv. Gastroenterol..

[53] Da Rio L., Spadaccini M., Parigi T.L., Gabbiadini R., Dal Buono A., Busacca A., Maselli R., Fugazza A., Colombo M., Carrara S. (2023). Artificial intelligence and inflammatory bowel disease: Where are we going?. World J. Gastroenterol..

