# Supplementary material for: Biologic Therapy and Surgical Management in Crohn’s Disease: Postoperative Outcomes and Biologic Management Patterns in a Retrospective Cohort Study
Source: Medicina (Kaunas). 2026 May 8;62(5):917. doi: 10.3390/medicina62050917 (PMC13209127; doi:10.3390/medicina62050917)
Supplement: Supplementary file 1 [file medicina-62-00917-s001.zip › medicina-4263266-supplementary.pdf]

Supplementary Materials

Supplementary Table S1. Anatomical disease features and surgical procedures (N = 60).

Variable	Category	n (%; 95% CI)
**Anatomical disease features**	Intra-abdominal abscess	23 (38.3%; 95% CI: 26.0–50.6)
	Intra-abdominal fistulas	20 (33.3%; 95% CI: 21.4–45.2)
	Ileal stricture	37 (61.7%; 95% CI: 49.4–74.0)
	Colonic stricture	13 (21.7%; 95% CI: 11.3–32.1)
	Rectal stricture	2 (3.3%; 95% CI: 0–7.9)
	Long (> 5 cm) or multiple strictures	25 (41.7%; 95% CI: 29.2–54.2)
	Short strictures (< 5 cm)	23 (38.3%; 95% CI: 26.0–50.6)
**Surgical procedures**	Ileocecal resection	31 (51.7%; 95% CI: 39.1–64.3)
	Right hemicolectomy	9 (15.0%; 95% CI: 6.0–24.0)
	Segmental colonic resection	6 (10.0%; 95% CI: 2.4–17.6)
	Left hemicolectomy	6 (10.0%; 95% CI: 2.4–17.6)
	Enterectomy (isolated small bowel)	5 (8.3%; 95% CI: 1.3–15.3)
	Total colectomy / proctocolectomy	5 (8.3%; 95% CI: 1.3–15.3)
	Subtotal colectomy	1 (1.7%; 95% CI: 0–5.0)
	Rectal / rectosigmoid resection	5 (8.3%; 95% CI: 1.3–15.3)
	Abscess drainage only	3 (5.0%; 95% CI: 0–10.5)
	Stricturoplasty (Heineke–Mikulicz / Michelassi)	2 (3.3%; 95% CI: 0–7.9)

Note: Variables are non-mutually exclusive and may overlap. Clinical features such as strictures and penetrating disease behavior were recorded descriptively and could coexist within the same patient. Most intra-abdominal abscesses were managed during bowel resection, with few cases requiring isolated drainage. Strictures were recorded descriptively and could overlap with penetrating disease behavior.

Supplementary Table S2. Univariate analysis of factors associated with combined resection procedures (N = 60).

Variable	*Combined resection procedures Yes (n=36)*	*Combined resection procedures No (n=24)*	*p-value*
Intra-abdominal Abscesses	*23 (63.9%)*	*0 (0%)*	***< 0.001***
Intra-abdominal Fistulas	*19 (52.8%)*	*1 (4.2%)*	***< 0.001***
Hypoalbuminemia	*13 (36.1%)*	*2 (9.1%)*	***0.031***
Elevated CRP (>50 mg/L)	*27 (75.0%)*	*6 (27.3%)*	***0.001***
Biologic Intensification	*17 (47.2%)*	*4 (16.7%)*	***0.026***
Preoperative Biologic Exposure	*21 (58.3%)*	*20 (83.3%)*	*0.051*
Multiple Strictures (>5 cm)	*14 (38.9%)*	*11 (45.8%)*	*0.606*
Prior Biologic Lines (median, range)	*1 (0–4)*	*1 (0–2)*	*0.236*

Note: Significant p-values (<0.05) are highlighted in bold. Biologic intensification is a postoperative variable and is presented for exploratory association only; it should not be interpreted as a predictor of intraoperative surgical decisions.

Supplementary Table S3. Univariate analysis of predictors associated with ileostomy and colostomy (N = 60).

**Variable**	**Ileostomy (n = 18)**	**No Ileostomy (n = 42)**	***p*-value**	**Colostomy (n = 8)**	**No Colostomy (n = 52)**	***p*-value**
**Preoperative Predictors**						
**Intra-abdominal Fistulas**	10 (55.6%)	10 (23.8%)	**0.034**	6 (75.0%)	14 (26.9%)	**0.013**
**Perianal disease**	3 (16.9%)	9 (21.4%)	1.000	6 (75.0%)	6 (11.5%)	**< 0.001**
**Preoperative Hypoalbuminemia**	7 (38.9%)	8 (20.0%)	0.194	5 (62.5%)	10 (20.0%)	**0.022**
**Intra-abdominal abscess**	10 (55.6%)	13 (31.0%)	0.089	6 (75.0%)	17 (32.7%)	**0.045**
**Surgical Urgency**	7 (38.9%)	9 (21.4%)	0.207	2 (25.0%)	14 (26.9%)	1.000
**CRP > 50 mg/L**	12 (66.7%)	21 (52.5%)	0.396	7 (87.5%)	26 (52.0%)	0.121
**Previous Surgeries**	9 (50.0%)	18 (42.9%)	0.778	5 (62.5%)	22 (42.3%)	0.448
**Preoperative anti-TNF exposure**	8 (44.4%)	10 (23.8%)	1.000	2 (25.0%)	6 (11.5%)	0.264

**Operative Characteristics**						
**Left Hemicolectomy**	0 (0.0%)	6 (14.3%)	0.165	5 (62.5%)	1 (1.9%)	**< 0.001**
**Ileocecal Resection**	9 (50.0%)	22 (52.4%)	1.000	1 (12.5%)	30 (57.7%)	**0.024**
**Right Ileo-hemicolectomy**	2 (11.1%)	7 (16.7%)	0.710	0 (0.0%)	9 (17.3%)	0.339
**Segmental Colonic Resection**	2 (11.1%)	4 (9.5%)	1.000	0 (0.0%)	6 (11.5%)	0.585
**Proctocolectomy/Total Colectomy**	3 (16.7%)	2 (4.8%)	0.154	1 (12.5%)	4 (7.7%)	0.524
**Subtotal Colectomy**	1 (5.6%)	0 (0.0%)	0.300	0 (0.0%)	1 (1.9%)	1.000
**Enterectomy**	1 (5.6%)	4 (9.5%)	1.000	2 (25.0%)	3 (5.8%)	0.128
**Rectal/Rectosigmoid Resection**	3 (16.7%)	2 (4.8%)	0.154	2 (25.0%)	3 (5.8%)	0.128

*Note**:** Data are presented as n (%). Significant p-values (<0.05) are highlighted in bold. Percentages are calculated relative to the total number of patients within each outcome group. The "No ileostomy" group includes patients with colostomy, and the "No colostomy" group includes patients with ileostomy. Higher proportions of perianal disease and left hemicolectomy were observed among patients undergoing colostomy, whereas ileocecal resections were more frequent in patients without colostomy.*

Supplementary Table S4. Correlation between Clinical and Therapeutic Factors and the Achievement of Postoperative Biological Response at 6 Months.

Variable	Postoperative biological response (Yes)	Postoperative biological response (No)	*p*-value
**Combined resection procedures (Yes)**	8 (66.7%)	28 (58.3%)	0.746
**Biologic intensification (Yes)**	3 (25.0%)	18 (37.5%)	0.513
**Preoperative anti-TNF (Yes)**	4 (33.3%)	24 (50.0%)	0.349

Note: Postoperative biological response is defined as the simultaneous normalization of hemoglobin (absence of anemia), serum albumin (>3.5 g/dL), and C-reactive protein (CRP ≤ 5 mg/L). Percentages are calculated within each column. Variables are not mutually exclusive.

Supplementary Table S5. Cumulative Surgical Burden and Longitudinal Evolution (N = 60).

Variable	Category	n (%; 95% CI)
**Primary Surgery**	Single surgical intervention (no reintervention required)	32 (53.3%; 95% CI: 40.7–65.9)
	Required ≥1 additional surgery	28 (46.7%; 95% CI: 34.1–59.3)
**Second Surgery Indication (n = 28)**	Stoma reversal (± additional resection)	19 (67.9%; 95% CI: 50.6–85.2)
	Reinterventions for unfavorable disease course*	9 (32.1%; 95% CI: 14.8–49.4)
**Third Surgery (n = 60)**	Required third intervention	6 (10.0%; 95% CI: 2.4–17.6)
**Fourth Surgery (n = 60)**	Required fourth intervention	2 (3.3%; 95% CI: 0–7.9)

Note: Reinterventions for unfavorable disease course include additional resections without restoration of bowel continuity and reoperations performed for postoperative complications.
